# Tuberculosis and coronavirus disease 2019 coinfection

**DOI:** 10.1590/0037-8682-0671-2020

**Published:** 2020-11-06

**Authors:** Daniel Oliveira Pinheiro, Mariana Santos Leite Pessoa, Carla Franco Costa Lima, Jorge Luis Bezerra Holanda

**Affiliations:** 1 Hospital Geral de Fortaleza, Departamento de Radiologia, Fortaleza, CE, Brasil.

Little is known about the relationship between coronavirus disease 2019 (COVID-19) and tuberculosis (TB). Recent studies have indicated that individuals with either latent or active TB may be more susceptible to infection with severe acute respiratory syndrome coronavirus 2 (SARS-CoV-2), and the progression of the disease caused by this virus may be faster and more severe than in patients without TB^1^.

Although these cases have rarely been reported in current literature^2^, studies have indicated that the isolation of TB cases can be an important measure to minimize the occurrence of severe cases of COVID-19 and associated hospitalizations^3^.

A 68-year-old male patient, who was diabetic and hypertensive and had chronic liver disease secondary to schistosomiasis, sought medical assistance, presented with dyspnea, fever, and cough for 1 week and was diagnosed with SARS-CoV-2 infection via the immunochromatographic solorological rapid test.

**FIGURE 1:**
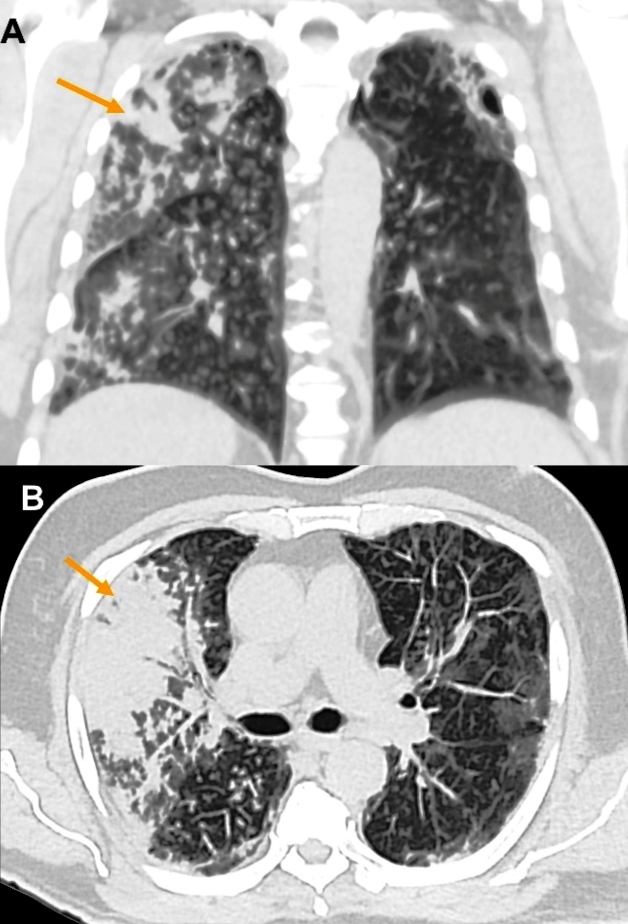
Chest CT in lung window, coronal (A) and axial (B) slices, show multiple centrilobular opacities, such as small nodules and a “tree-in-bud” pattern, with areas of confluence of consolidating opacities (arrows), affecting the right lung. In the left lung, opacities with ground-glass attenuation are observed, notably peripherally, with associated fine reticular opacities.

**FIGURE 2:**
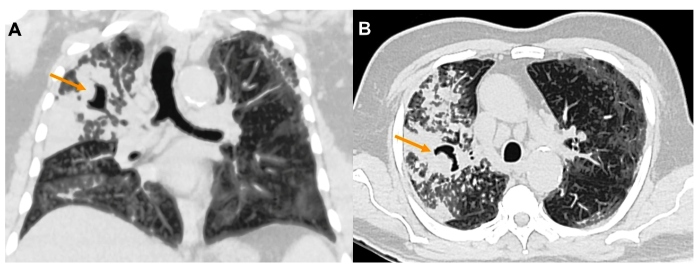
Chest CT in lung window, coronal **(A)** and axial **(B)** slices, show multiple centrilobular opacities, such as small nodules and a “tree-in-bud” pattern, with areas of confluence of consolidating opacities, some with areas of cavitation in the upper right lobe (arrows).

**FIGURE 3:**
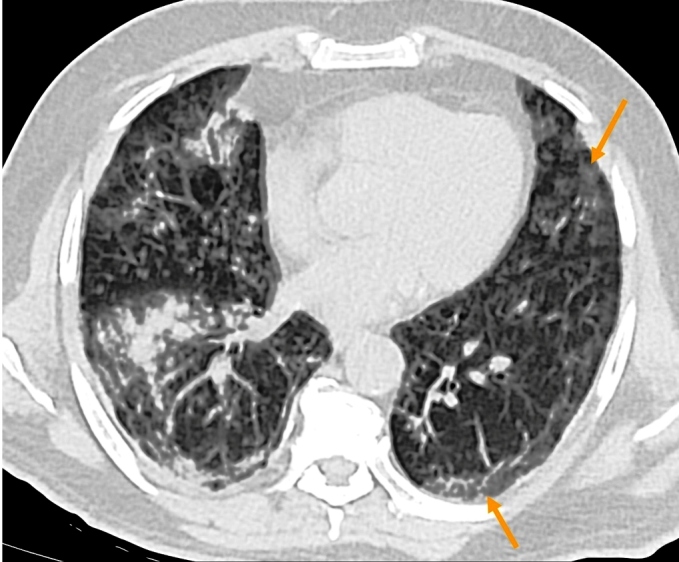
An axial slice chest CT in lung window shows multiple centrilobular opacities, such as small nodules and a “tree-in-bud” pattern, with areas of confluence of consolidating opacities, affecting the right lung. In the left lung, opacities with ground-glass attenuation are observed, notably peripherally (arrows), with associated discreet reticular opacities.

Computed tomography (CT) of the chest showed changes suggestive of bronchogenic dissemination of the infection, providing a diagnostic hypothesis of an infectious/inflammatory process of granulomatous etiology (Figure 1 and Figure 2). Discrete ground-glass opacities were also noted, suggestive of an associated viral infection (Figure 3). 

Special attention was given to the risk of coinfection with TB, given that Brazil has an extensive number of TB cases and is currently one of the epicenters of the COVID-19 pandemic. Thus, GeneXpert MTB/RIF was performed, which presented a positive result for sensitivity to rifampicin as well as positive Acid-Alcohol Resistant Bacillus (BAAR) results in the three sputum samples acquired. The patient was referred for specific isolation during his hospitalization, with a coinfection diagnosis of SARS-CoV-2 and *Mycobacterium tuberculosis*.
